# Logging Affects Fledgling Sex Ratios and Baseline Corticosterone in a Forest Songbird

**DOI:** 10.1371/journal.pone.0033124

**Published:** 2012-03-14

**Authors:** Rhiannon Leshyk, Erica Nol, Dawn M. Burke, Gary Burness

**Affiliations:** 1 Environmental and Life Sciences Graduate Program, Trent University, Peterborough, Ontario, Canada; 2 Biology Department, Trent University, Peterborough, Ontario, Canada; 3 Ontario Ministry of Natural Resources, London, Ontario, Canada; Monash University, Australia

## Abstract

Silviculture (logging) creates a disturbance to forested environments. The degree to which forests are modified depends on the logging prescription and forest stand characteristics. In this study we compared the effects of two methods of group-selection (“moderate” and “heavy”) silviculture (GSS) and undisturbed reference stands on stress and offspring sex ratios of a forest interior species, the Ovenbird (*Seiurus aurocapilla*), in Algonquin Provincial Park, Canada. Blood samples were taken from nestlings for corticosterone and molecular sexing. We found that logging creates a disturbance that is stressful for nestling Ovenbirds, as illustrated by elevated baseline corticosterone in cut sites. Ovenbirds nesting in undisturbed reference forest produce fewer male offspring per brood (proportion male = 30%) while logging with progressively greater forest disturbance, shifted the offspring sex ratio towards males (proportion male: moderate = 50%, heavy = 70%). If Ovenbirds in undisturbed forests usually produce female-biased broods, then the production of males as a result of logging may disrupt population viability. We recommend a broad examination of nestling sex ratios in response to anthropogenic disturbance to determine the generality of our findings.

## Introduction

Natural selection should favour those parents who invest equally in both sexes [1]. However, when the quality of the investing parent is lowered, equal investment in both sexes may not result in the highest fitness [2]. Trivers and Willard (1973) predicted that parents should adjust the sex ratio of offspring to match the ability of a parent to invest. Specifically, mothers in better condition should produce more sons, while mothers in relatively poor condition should produce more daughters [3]. Production of sons should only be favoured if there is a reasonable probability that these sons will be able to out-compete conspecifics for mates [4], [5]. Since, in most monogamous and polygynous species males compete for females, females will nearly always reproduce while males may not be chosen for mating or may be unable to compete against other, better quality males [3]. Studies with a variety of vertebrates (feral horses [*Equus caballus*]: [6]; European starlings [*Sturnus vulgaris*]: [7]; Peafowl [*Pavo cristatus*]: [8]; White-crowned Sparrows [*Zonotrichia leucophrys*]: [9]) have shown that reducing maternal quality causes the sex-ratio to be shifted towards female offspring. Production of female offspring is linked to environmental quality [10], [11], a factor which strongly influences maternal condition.

Anthropogenic disturbances modify the landscape and consequently alter environmental quality. Such disturbances may cause stress in wildlife, resulting in elevated levels of the stress hormone corticosterone (e.g. tourism [12], pollution [13], habitat removal/disturbance [12], [13]). Since corticosterone can be quantified relatively easily from blood samples, and because of the link to quality and fitness [14], [15], it has become a tool used by conservation biologists in assessing population health in relation to environmental challenges [16]. Human landscape changes may thus have the ability to skew sex ratios if disturbance translates into a change in habitat quality.

Many neotropical migrant songbirds rely on forested habitats in temperate regions for successful breeding. Forest harvesting subjects breeding birds to conditions which can cause physiological stress [13], [17]–[19]. The degree to which forest environments are modified depends on the logging prescription and forest stand characteristics. For example, with group-selection silviculture (GSS), small clear-cuts (gaps, 0.03–0.07 ha) are made throughout a stand with the quantity and layout of the gaps differing across logging prescriptions. This method creates larger canopy gaps than those created through the single-tree selection system, a method applied frequently in hardwood forests [20]. GSS is favoured by some forest managers because it allows for more light to access seedlings for a longer period of time. With increased desire to shift from conventional forest harvesting (i.e., chainsaws and skidders) to more mechanized systems (i.e., feller-bunchers), and growing pressure to reduce operating costs [21], methods like GSS will likely be favoured for their efficiency.

The Ovenbird, a small (ca. 20 g) socially monogamous, sexually-monomorphic, forest interior, neotropical migrant passerine [22] is potentially, adversely impacted by GSS. Ovenbirds typically breed in large tracts of continuous forest with dense canopy cover and thick leaf litter that promotes their invertebrate prey [23], two characteristics that are disturbed or removed as a result of GSS [24]. Additionally, Ovenbirds have previously been shown as an informative species for physiology studies, making them an appropriate choice for the current study [25]. We compared the effects of no harvesting, to moderate and heavy GSS on offspring sex ratios and nestling plasma baseline corticosterone in Algonquin Provincial Park, Ontario, Canada. We do not know how Ovenbird sons and daughters differ in reproductive cost. Therefore, we predicted that broods from harvested sites would be female-biased, because poor-quality habitats may lack the resources necessary to produce the more risky males [10], [11]. In undisturbed reference sites we predicted that offspring sex ratios would not differ from parity [1]. Finally, we predicted nestling Ovenbirds from harvested sites would have elevated baseline corticosterone levels.

## Materials and Methods

### (a) Study area and treatments

The study was conducted in the southern portion of Algonquin Provincial Park, Ontario, Canada (45°32′N, 78°36′W) between May and August of 2008 and 2009. All sites were located in upland hardwood forest dominated by sugar maple (*Acer saccharum*; ∼72%). American beech (*Fagus grandifolia*), yellow birch (*Betula alleghaniensis*), eastern hemlock *(Tsuga canadensis)*, and a very small proportion of black cherry (*Prunus serotina*; ∼1%) made up the remainder of the forest tree composition.

**Figure 1:**
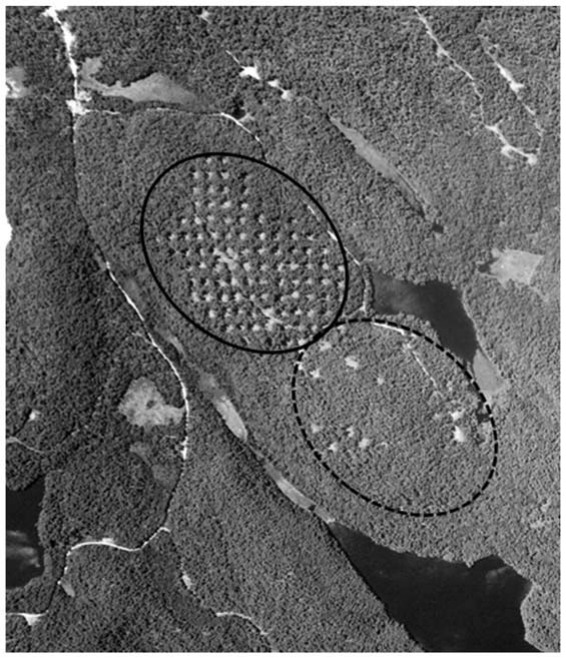
Aerial image of two forest stands cut with group-selection silviculture (GSS) in Algonquin Provincial Park. Heavy group-selection is outlined with a solid line in the upper left and Moderate group-selection is outlined with a dotted line in the bottom right. Undisturbed sites are similar to the unharvested forest in the upper right of the image (Source: Algonquin Forestry Authority).

The study consisted of three harvesting treatments of differing intensity of disturbance: undisturbed, moderate disturbance and heavy disturbance. Undisturbed sites were chosen within the park's designated development and natural environment zones, or nature reserve zones. These areas do not currently permit harvesting and none has occurred within the last 60 years. Moderate disturbance sites had a mixture of small (0.03 ha) or large (0.07 ha) gaps placed strategically next to at least one mature shade sensitive tree for a total of 10–12 gaps per stand. At least 5 small gaps were placed next to yellow birch seed trees and 5 large gaps placed next to black cherry seed trees. The remainder of the stand was cut to a basal area of 18–20 m^2^ ha^−1^ following the single-tree guidelines of the Province of Ontario, Canada [26]. Heavy disturbance sites had medium-sized, 0.05 ha gaps placed uniformly in a grid 50 m from each gap centre. All harvesting was limited to the gaps and no harvesting, with the exception of skid trails for travel, was performed in the forested matrix. In the heavy disturbance sites, approximately 4 gaps of trees per hectare were removed, with an average harvested site (20 ha) containing 80 gaps (Figure 1). We used three replicate stands per each silvicultural treatment and per reference conditions, for a total of 9 sites. Sites ranged from 25–44 ha (heavy (25±10 ha [mean±SD]), moderate (35±6 ha), undisturbed (43±1 ha) and all harvesting occurred between August 2006 and February 2007. All sites were embedded in a mostly continuous forested landscape.

### (b) Field procedures

Nest searching began when the first female Ovenbird was heard in any of the study sites; in both years, this occurred approximately in the third week of May. Nests were located by using visual or auditory cues such as female ovenbirds carrying nesting material, or defensive calls from either parent when in close proximity to the nest or by incidentally flushing incubating females. Nests were visited every 2–5 days during building and incubation to monitor status, and every day closer to the predicted hatch date (near 12 d of incubation). Nests were checked from a distance with binoculars, unless verifying the date of hatch in which case incubating females were usually flushed. Disturbance was therefore kept to a minimum and all nests were disturbed approximately equally.

Following hatching, nests were revisited once when the nestlings were 6–7 days of age, approx. 1–2 days before fledging. Individuals were weighed, measured and fitted with a unique combination of colour bands and an aluminum identification band. Two 20–50 µl blood samples were collected from each nestling within 3-min of disturbance of the nest site. Blood extraction within a 3-minute time-limit best represents a baseline sample because there is insufficient time for corticosterone to increase as a result of the acute stress response [27]. One sample (10 µl) was mixed in the field with 1 ml of lysis buffer (0.1M Tris, 0.1M EDTA, 0.1M NaCl, 0.3M n-laurylsarcosine, pH of 7.5) for later molecular sexing. The remaining sample was stored on ice for up to 4 hours before being centrifuged to remove the plasma. The blood and lysis buffer mixture, and plasma samples were initially stored at −20°C, and transferred to −80°C. All practices conformed to the rules and regulations of the Trent Animal Care Committee under Permit 08042 and samples were collected under a Canadian Wildlife Service collection permit CA 0202.

### (c) Laboratory analysis

Total corticosterone concentration was determined using a corticosterone I^125^ double-antibody radio-immunoassay (RIA) kit (MP Biomedicals, Solon, OH, USA) following manufacturer's instructions with modifications as per [28]. Samples were run in duplicate and inter-assay variance was 9.5%.

We sexed nestlings using primers P8 and CW that amplify a gene from the CHD locus, located on both sex chromosomes [29]. Briefly, DNA was extracted using DNeasy kits (Qiagen, Mississauga, ON, Canada) following manufacturer's protocols. DNA was extracted into a final volume of 100 µl and frozen at −20°C until needed. Ovenbird nestlings were sexed using a polymerase chain reaction (PCR) amplification procedure previously outlined in [29]. PCR amplification was carried out in a Px2 Thermal Cycler (Thermo Electron Corporation, Waltham, MA, USA). PCR reaction conditions were as follows: 1.0× buffer, 2.0 mM MgCl_2_, 0.2 mM dNTPs, 1.6 µM CW/P2 primers, 1.25 U Taq polymerase (Invitrogen Canada Inc., Burlington, ON, Canada) and 150–250 ng extracted DNA; for a final volume of 25 µl. 10 µl PCR product was separated by gel electrophoresis at 100 V for 90 minutes on a 3% agarose gel, run in standard TAE buffer, then visualized by ethidium bromide staining. This percentage of agarose was used to aid in band separation. In addition to nestlings, blood samples collected from adults who had been sexed in the field by presence of either a cloacal protuberance (male) or brood patch (female) were molecularly sexed using the same procedure listed above to verify visual sexing techniques.

### (d) Statistical Analyses

Statistical tests were performed using R 2.11.1 (The R Foundation for Statistical Computing, 2010). Data were tested for the assumptions associated with linear models where appropriate and *p* = 0.05 was set as the level of significance for all tests. Means are given with standard error of the mean (SE).

One nest was excluded from all analyses because it had an original clutch size of two eggs, one of which was later removed either by the parents or a predator. Daily survival rate (DSR) and nest success were calculated using the logistic exposure method [30]. We used PROC GENMOD (SAS Institute Inc. 2008) to calculate regression coefficients for logistic exposure models. Akaike's information criterion (AIC), adjusted for small sample sizes (AICc), was used to determine the best-supported models from a suite of candidate models. Models with ΔAIC values <2 were considered to have the most support. Importance weights (sum of the Akaike weights) were calculated for each parameter within the best model set to determine which should be included in the final model. To account for model uncertainty, model averaged parameter estimates were calculated for all parameters in the top model set.

Models were constructed a priori and included linear, quadratic (x+x_2_), and cubic (x+x_2_+x_3_) effects of nest age, and linear and quadratic (x+x_2_) effects of annual Julian date (hereafter, “Julian date”; Grant et al. 2005) [31]. The quadratic effect of date is plausible if we consider that survival can vary relative to temporal fluctuations in predator abundance and food availability. Furthermore, the quadratic and cubic effects of nest age take into consideration that survival can be dependent on nest stage with survival probability differing between incubation, hatching, and chick rearing. In addition to time effects, treatment (TREATMENT: heavy, moderate, or undisturbed) was included as a categorical variable in models predicting nest survival. In addition to candidate models, a constant survival model, and a global model comprised of all parameters, were constructed. Candidate models were constructed from combinations of the following parameters: linear (AGE), quadratic (AGE^2^), and cubic (AGE^3^) nest age, and linear (JULDATE), and quadratic (JULDATE^2^) Julian date, YEAR, TREATMENT and the interaction between linear nest age and Julian date (AGE×JULDATE). Daily survival rate and nest success (NS) were calculated using top model parameter estimates (β_0_ and β_1x_) after Shaffer (2004) [30].

Offspring sex ratio was analyzed using two common methods. A general linear mixed model (the function “lme” in the package *nlme* for R; the R Core team 2010) was run to determine if the proportion of males per brood (male sex ratio; calculated as a proportion of male nestlings per brood) differed across treatments. A generalized linear mixed model with binomial errors (the function “lmer” in the package *lme4* for R; the R Core team 2010) was used to determine if the probability of obtaining a male nestling within a brood differed across treatments. The results were qualitatively the same, giving the same significance for both analyses. We present the results of the general linear mixed model because they are intuitively easier to interpret. We present results of two different models, one containing treatment, year and complete (complete or incomplete clutch) variables and a minimal model containing only Treatment. Site was added as a random effect to all models. To determine if male sex ratio differed from parity we used a G-test of contingency (the function “chisq.test”: the R Core team 2011).

We analyzed nestling baseline corticosterone levels and body condition using a general linear mixed model (the function “lme” in the package *nlme* for R; the R Core team 2010). Nestling corticosterone values was log10 transformed (Kolmogorov-Smirnov Test: d = 0.08, *P*>0.20) to improve spread of residuals. Nestling body condition was quantified as the residuals from a regression of individual mass on tarsus length [32] but we also present the results of analyses of mass and mass with tarsus as a covariate for comparison purposes. We chose to represent body condition as the residuals of a regression of mass on tarsus because it is a measure commonly used throughout literature and it has been shown previously that this measure satisfies critical assumptions [32]. For analysis of nestling corticosterone, treatment, body condition, sex, year and the interaction treatment×sex were included in the initial model but the interaction term was later removed as it was not significant. Initial inclusion of the treatment×sex interaction makes biological sense as either sex may respond differently to stressors (i.e. logging). Nest ID was added as a random effect to control for relatedness between siblings and was nested within Site. Site was included as a random effect to account for potential differences between sites within treatments. A Tukey's HSD test was used to identify significant differences between pairs of treatments. Nestling corticosterone results are presented as back-transformed values.

## Results

During 1549 search hours over two years of study, we found 70 Ovenbird nests. More nests were found in the undisturbed treatment (n = 31) than either the moderate (n = 23) or heavy treatments (n = 15). The number of nests discovered/search hour for the undisturbed treatment was the same as for the moderate treatment (0.05), suggesting a similar abundance of nesting Ovenbird pairs in these treatment types. In contrast, the heavy treatment probably had fewer nesting Ovenbird pairs than the other treatments, as evidenced by both the lower number of nests discovered per hour of searching (0.03) and lower total number of nests found. The primary cause of nest failure was predation (55.7% of all nests) but a considerable number were abandoned (18.6%). Moreover, baseline corticosterone samples (within 3 minutes) could not be obtained for all nestlings. As a result, 106 nestlings from 23 nests were used in analyses of brood sex ratios, while 55 nestlings from 17 nests were used in corticosterone analysis. Sixty-two nests were used in calculations of nest success and daily survival rate (DSR).

The global model fit the observed data well (*X*_2_ = 258.48, df = 258, *P* = 0.52). The variable Treatment was not included in the top model set and was one of the least supported models overall (Table 1). Nest success was best explained by AGE and JULDATE^2^ (Table 1) with nest success declining both as a function of date and age. Nest success ranged from 50.2% (heavy) to 55.1% (moderate). Nest success in the undisturbed stands was intermediate (52.9%).

**Table 1:** The top logistic exposure models of daily survival rate of Ovenbird nests in Algonquin Provincial Park in 2008 and 2009 as chosen using AIC model selection corrected for small sample size (AICc).

Model	K	Log(L)	AICc	ΔAICc	ωi
JULDATE^2^	3	−85.77	177.57	0.000	0.1094
**AGE+JULDATE^2^**	**4**	**−85.51**	**179.07**	**1.504**	**0.0516**
AGE+JULDATE	3	−86.76	179.55	1.981	0.0406
JULDATE2+YEAR	4	−85.76	179.56	1.995	0.0403
CONSTANT	1	−91.89	185.79	8.224	0.0018
GLOBAL	10	−84.21	187.12	9.550	0.0009
TREATMENT	3	−91.39	188.82	11.250	0.0004

Models with ΔAICc values <2 were considered the most supported. Treatment was not a well supported model as it failed to appear in any of the top models. The chosen model is displayed in bold.

Thirteen clutches had the same number of nestlings as eggs laid (primary sex ratio) and ten had a lower number of nestlings than eggs laid (fledgling sex ratio). Incomplete clutches resulted from unhatched eggs or nestling mortality and their subsequent removal from the nest. Because clutch reductions occurred in the time between nest checks, the mechanism responsible was usually undetermined. There was no significant difference in sex ratio between complete and incomplete nests (*P* = 0.19, Table 1), so both were pooled for analysis of fledging sex ratio.

**Figure 2:**
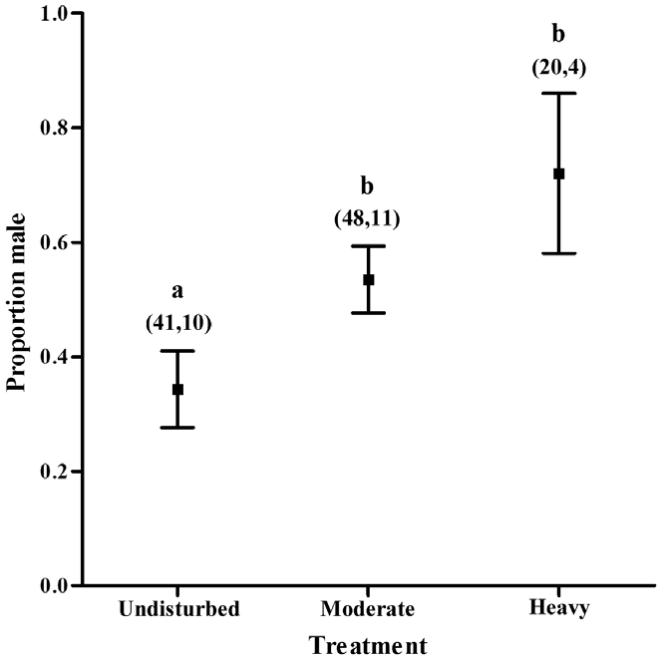
The proportion of males within a brood within a given treatment. Means are presented with standard error, and the number of nestlings and the number of broods within each treatment respectively are in parentheses. Different letters indicate significant differences.

**Figure 3:**
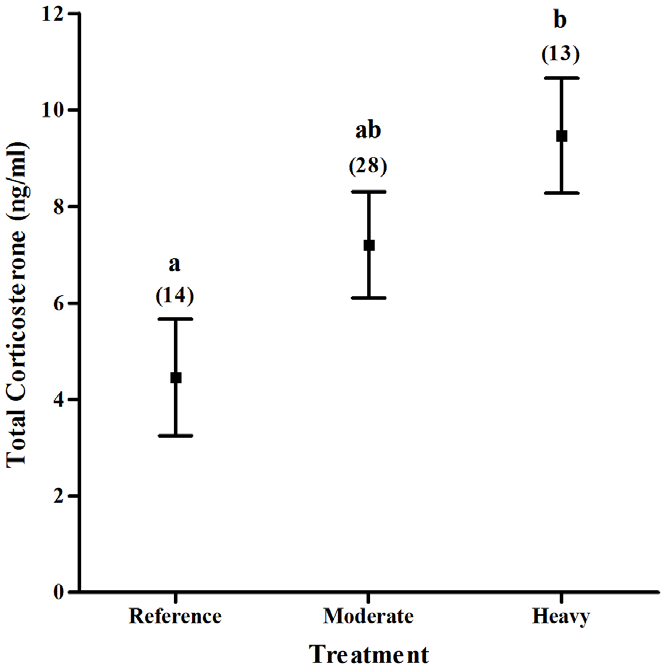
Effect of harvesting treatment on the mean corticosterone levels in broods of Ovenbird nestlings. Brood corticosterone levels were significantly higher in the Heavy logging treatment than the Undisturbed logging treatment (*P* = 0.003). The number of nestlings and the number of broods within each treatment respectively are in parentheses. Means and SE are presented. Different letters indicate significant differences using Tukey's unequal N HSD post hoc test.

Fledging sex ratio did not deviate from parity when all treatments were pooled (χ^2^ = 26.193, *P* = 0.34) but varied significantly among harvesting treatments (*P* = 0.031, Table 2). Broods were significantly more male-biased in the heavy treatment than the undisturbed but broods from the moderate treatment did not differ from either the heavy or undisturbed treatments (Figure 2 and Table 1). Male sex ratio did not differ across years (*P* = 0.91, Table 2). Nestling baseline corticosterone levels differed significantly across treatments (*P*<0.05, Table 3), but not across years (*P* = 0.25, Table 3) and nestlings from heavily harvested stands had more than twice the level of corticosterone than individuals from undisturbed stands (post hoc *P* = 0.003, Figure 3). Nestling corticosterone levels in moderately harvested stands were higher than those in the undisturbed stands but did not differ from those in the heavy treatment (Table 3). Nestling corticosterone values did not differ significantly between male and female nestlings (*P* = 0.33, Table 2; male = 8.46±4.4 ng/ml, female = 7.24±5.3 ng/ml) and there was no relationship between nestling body condition (calculated as the residuals of mass against tarsus length; *r* = 0.88, *P*<0.0001) and baseline corticosterone levels (*P* = 0.729, Table 3). As well, nestling body condition did not differ across treatments (*F*_2,92_ = 0.24, *P* = 0.80), sexes (*F*_1,92_ = 0.02, *P* = 0.90) or years (*F*_1,92_ = 0.01, *P* = 0.93; Table 4). Furthermore, neither nestling mass (*F_2,96_* = 0.51, *P* = 0.60) nor nestling mass with tarsus as a covariate (*F_2,96_* = 2.18, *P* = 0.12) differed across treatments (Table 4).

**Table 2:** Results of a linear mixed model for nestling male sex ratio, represented as the proportion of male nestlings within a given brood.

	*F*-value	d.f.	*P*-value
Full Model			
Treatment	4.03	2	0.033
Complete	1.85	1	0.187
Year	0.02	1	0.910
Minimal Model			
Treatment	4.05	2	0.031
Tukey Contrasts	Estimate	s.e.	*P*-value
Heavy - Undisturbed	2.00	0.71	0.013
Moderate - Undisturbed	0.77	0.52	0.300
Moderate - Heavy	−1.22	0.70	0.183

Both the full model containing, treatment, complete (whether the brood size equalled the clutch size), and year, and a minimal model containing just treatment are presented. Pairwise significances were determined using Tukey's post-hoc analysis, estimate, standard error and p-values are presented.

**Table 3:** Results of a linear mixed model for nestling baseline corticosterone.

	*F*-value	d.f.	*P*-value
Full Model			
Treatment	6.07	2	0.005
Body Condition	0.12	1	0.729
Year	1.34	1	0.253
Sex	1.05	1	0.311
Treatment×Sex	2.32	2	0.110
Minimal Model			
Treatment	5.83	2	0.005
Tukey Contrasts	Estimate	s.e.	*P*-value
Heavy - Undisturbed	0.33	0.10	0.003
Moderate - Undisturbed	0.21	0.08	0.033
Moderate - Heavy	−0.12	0.09	0.347

Both the full model containing, treatment, body condition, year, sex and a treatment×sex interaction and a minimal model containing just treatment are presented. Pairwise significances were determined using Tukey's post-hoc analysis, estimate, standard error and p-values are presented.

**Table 4:** Results of a linear mixed model for nestling body condition.

	*F*-value	d.f.	*P*-value
Full Model			
Treatment	0.24	2	0.785
Sex	0.02	1	0.895
Year	0.01	1	0.928
Treatment×Sex	0.45	2	0.640
Minimal Model			
Treatment	0.25	2	0.779
Tukey Contrasts	Estimate	s.e.	*P*-value
Heavy - Undisturbed	−0.09	0.33	0.961
Moderate - Undisturbed	−0.11	0.28	0.913
Moderate - Heavy	−0.03	0.33	0.997

Both the full model containing, treatment, sex, year and a treatment×sex interaction and a minimal model containing just treatment are presented. Pairwise significances were determined using Tukey's post-hoc analysis, estimate, standard error and p-values are presented.

## Discussion

Logging, specifically heavy GSS, significantly increased baseline corticosterone in nestling Ovenbirds. We did not find a relationship between corticosterone and either body condition or mass, indicating that the observed increase in baseline corticosterone in logged sites was not likely the result of reduced food abundance, a mechanism previously known to elevate baseline corticosterone in nestling birds [33]. Elevation of baseline corticosterone is consistent with other studies that have shown elevated levels in response to forest harvesting [17]–[19] but contrasts with a specific study conducted on Ovenbirds in fragmented boreal habitats [25]. Mazerolle and Hobson (2002) found higher indices of stress in adult male Ovenbirds in contiguous forest patches but not in fragmented areas. While the results of this study are interesting, it is difficult to interpret their results in the context of our study as those authors measured both a different life stage and a different physiological index. While we investigated changes in stress hormones in developing young they measured haematological indices in adult males. Overall, chronic environmental stress in developing young as shown by elevated baseline corticosterone levels can have many sub-lethal detrimental effects including reduced immunocompetence [34], growth [34]–[37], cognitive ability [38] and dominance in adulthood [39].

We also detected significant nestling sex-ratio biases in logged forests. Under regular undisturbed conditions, broods contained more female nestlings, while logging shifted the offspring sex ratio towards males. It appears that logging, particularly heavy GSS, creates a more stressful environment that corresponds to an increased proportion of male offspring within a nest. However, while our patterns are strong and consistent between our two years of study, we acknowledge that our sample sizes were small and thus our results must be interpreted with caution.

Biases in avian sex ratios can occur at egg laying (primary sex ratio), hatching (secondary sex ratio) or fledging (tertiary sex ratio). Although some studies have shown that females can bias primary sex ratios in an adaptive way [40], the nature of our sampling regime did not allow us to distinguish between the various forms of sex ratio bias. In our study we are referring to fledging sex ratio as we only have gender data from nestlings at fledging age, although sex ratios in nests with complete clutches (hatching sex ratio) were not significantly different from those in which one or several eggs were lost.

Given sex allocation theory, we expected, that under undisturbed conditions, broods would contain offspring at parity (50∶50 sex ratio) [1], because breeding Ovenbirds show high reproductive success in these conditions [41]. The finding of a female-skew of Ovenbird nestling sex ratios in the undisturbed sites is thus unexpected, and intriguing. We hypothesize that the female-skew potentially stems from an opposite skew in the sex ratio of the adult breeding population. Much literature on passerine adult sex ratios shows a skew towards males [42], which is a result of lower survival rates of female birds [43]–[48]. This lower survival of females appears to be a result of differences in the behaviour and physiology of the sexes. In many passerines, females are the sole nest incubators, having increased metabolic demands and predation risk [49], [50]. For example in Northern Wheatears (*Oenanthe oenanthe*), females were depredated during 20–40% of all nest predation events, a loss that, for the most part, the male population would not experience. In migratory species, females often disperse farther even though they are usually the smaller sex [51]–[53]. Finally, dominant males often exclude females from resources on the wintering grounds [54], a move that further reduces survival rates of females. Given the resulting male-bias in adult sex ratios in many species, it is possible that selection favours the production of female offspring in undisturbed conditions, to counteract losses that may arise post-fledging.

Evidence exists that female sex ratio biases can arise in absence of explanatory variables [55], but what is equally difficult to understand is why a disturbance like logging would shift the sex ratio towards males. In disturbed conditions (i.e. logged sites) where parental quality might be reduced due to a general reduction in habitat quality [56], we expected that if a sex-ratio bias did occur, it would be towards the production of females [2]. However, Ovenbirds in both GSS treatments (moderate and heavy) skewed offspring sex ratio towards males, relative to the undisturbed site, suggesting an increased sensitivity of nestling females. Our observation of male-biased broods in logged sites suggests a different tactic for maximizing fitness. Experimental studies of zebra finches (*Taeniopygia guttata*) have shown that breeding females, when provided with low quality or restricted diets prior to laying, bias broods toward males [57], [58]. This is presumably because under poor food conditions, chick mortality is biased toward females, while under good conditions it is biased toward males [58]. Additionally, female Blue Tit (*Parus caeruleus*) offspring also are more sensitive to mortality than males [59]. Accordingly, a female can increase her fitness returns under conditions of food restriction if she biases her brood toward males [57], [58]. We do not have data on sex-specific mortality in Ovenbirds. However, if female nestlings are likely to suffer disproportionate mortality under heavy group-selection, perhaps in the presence of competition between the sexes for suitable post-fledgling habitat [60], then adult females may maximize fitness returns by biasing brood sex ratios toward males.

Male-biased offspring sex ratios can occur in nests with enlarged broods [59] and in those subjected to brood parasitism [61], an obvious stressor for host nestlings. The stressful post-harvest environment created by GSS harvesting may also be responsible for the male shift in our study through female nestling mortality. While female nestling mortality remains a potential mechanism, it is unlikely given the lack of significant difference in sex ratios of nestlings from complete and incomplete clutches and in the lack of difference in body condition across treatments. We speculate that female Ovenbirds manipulate the sex ratio internally prior to laying the clutch.

Although the mechanism behind the observed skew is unknown, the potential consequences can be problematic from a population standpoint. As adult female passerines already experience higher mortality throughout their annual cycle, a mechanism that further reduces their numbers could be highly detrimental to the larger breeding Ovenbird population by significantly reducing the effective population size of this monogamous species. Our study differs from other experimental studies, in that we manipulated the whole environment in contrast to a single parameter (e.g. parental provisioning [38], brood size [57]) within or directly affecting a brood. Much still remains to be understood and future studies could be focused on Ovenbird behaviour, such as whether Ovenbirds forage within the gaps or avoid them for risk of predators. Additionally, future monitoring of offspring sex ratios in both disturbed and undisturbed habitats will help to elucidate the generality of our findings for other sexually monomorphic species.
